# Identification and precision therapy for three maturity-onset diabetes of the young (MODY) families caused by mutations in the HNF4A gene

**DOI:** 10.3389/fendo.2023.1237553

**Published:** 2023-08-29

**Authors:** Juan Zhang, Yanyan Jiang, Jianhua Li, Haiyin Zou, Li Yin, Yang Yang, Lei Yang

**Affiliations:** ^1^ Institute of Monogenic Disease, School of Medicine, Huanghuai University, Zhumadian, China; ^2^ Department of Scientific Research Section, Zhumadian Central Hospital, Affiliated Hospital of Huanghuai University, Zhumadian, China; ^3^ Department of Geriatric Endocrinology, The First Affiliated Hospital of Zhengzhou University, Zhengzhou, China; ^4^ Department of Emergency Intensive Care Unit, The First Affiliated Hospital of Zhengzhou University, Zhengzhou, China; ^5^ Department of Ultrasound Medicine, The 990th Hospital of The People’s Liberation Army, Zhumadian, China; ^6^ Zhumadian Key Laboratory of Chronic Disease Research and Translational Medicine, Institute of Cardiovascular and Cerebrovascular Diseases, School of Medicine, Huanghuai University, Zhumadian, China

**Keywords:** maturity-onset diabetes of the young, HNF4A gene, mutation, precision therapy, whole exome sequencing

## Abstract

**Background:**

Heterozygous pathogenic variants in HNF4A gene cause maturity-onset diabetes of the young type 1 (MODY1). The mutation carriers for MODY1 have been reported to be relatively rare, in contrast to the most frequently reported forms of MODY2 and MODY3.

**Methods:**

Whole exome sequencing (WES) and Sanger sequencing were performed for genetic analysis of MODY pedigrees. Tertiary structures of the mutated proteins were predicted using PyMOL software.

**Results:**

Three heterozygous missense mutations in the HNF4A gene, I159T, W179C, and D260N, were identified in the probands of three unrelated MODY families using WES, one of which (W179C) was novel. Cascade genetic screening revealed that the mutations co-segregated with hyperglycemic phenotypes in their families. The molecular diagnosis of MODY1 has partly transformed its management in clinical practice and improved glycemic control. The proband in family A successfully converted to sulfonylureas and achieved good glycemic control. Proband B responded well to metformin combined with diet therapy because of his higher body mass index (BMI). The proband in family C, with paternal-derived mutations, had markedly defective pancreatic β-cell function due to the superposition effect of T2DM susceptibility genes from the maternal grandfather, and he is currently treated with insulin. *In silico* analysis using PyMOL showed that the I159T and D260N mutations altered polar interactions with the surrounding residues, and W179C resulted in a smaller side chain.

**Discussion:**

We identified three heterozygous missense mutations of HNF4A from Chinese MODY families. Structural alterations in these mutations may lead to defects in protein function, further contributing to the hyperglycemic phenotype of mutation carriers.

## Introduction

Maturity-onset diabetes of the young (MODY) comprises approximately 1%–5% of all diabetes cases, demonstrating typical features including early onset, a familial history of diabetes inherited in an autosomal dominant pattern, and being non-insulin-dependent (1, 2). Currently, 13 genes on different chromosomes have been designated as MODY-causing genes (1), although mutations in other genes, such as *RFX6* and *APPL1*, have also been reported to be associated with MODY (3, 4). The most frequently reported subtypes are *GCK*-MODY (MODY2) and *HNF1A*-MODY (MODY3), which account for more than 80% of MODY cases, followed by *HNF4A*-MODY (MODY1), with an estimated proportion of 5% (4). MODY families have yielded the discovery of more than 100 variants in the HNF4A gene (5).

*HNF4A*-MODY is caused by variants in the gene encoding hepatic nuclear factor 4 (HNF4α), a member of the nuclear hormone receptor superfamily and a transcription factor, which exhibits predominant expression in the liver, gastrointestinal tract, kidney, and pancreatic islets. HNF4α contains a zinc finger DNA binding domain (DBD), which works as a homodimer binding DNA, and two transactivation domains (TADs), which are called AF1 (amino acids 1–24) and AF2 (amino acids 128–366) including the ligand binding domains (LBDs) and the dimerization interface (6–8). Heterozygous mutations in *HNF4A* causing MODY1 are attributed to the defect of HNF4α in the regulation of gene expression in pancreatic β-cell and its association with the coupling of metabolism secretion (6, 9).

Compared with type 2 diabetes mellitus (T2DM), *HNF4A*-MODY occurs at younger ages with lower hemoglobin A1c, body mass index (BMI), triglyceride levels, and a similar risk of microvascular complications. Roughly 50% of *HNF4A*-MODY patients have neonatal macrosomia, which paradoxically arises from transient hyperinsulinemic hypoglycemia (HH) during birth (10). Despite these distinguishing features, targeted sequencing of *HNF4A* based on clinical features may lead to misdiagnosis due to the absence of the classic presentation of *HNF4A*-MODY in some cases (11, 12). Individuals with *HNF4A*-MODY have increased sensitivity to sulfonylureas, and low doses of sulfonylureas produced better glucose control than metformin in those patients (3). Therefore, identifying patients suspected of having *HNF4A*-MODY to pursue etiology-based therapies will contribute to better glycemic control and cost-effectiveness.

In this study, we identified three heterozygous missense mutations in *HNF4A* co-segregating with the hyperglycemic phenotype using whole exome sequencing (WES), one of which was novel. In addition, integrated information on pedigree segregation, clinical data, and *in silico* simulation analysis was used to evaluate the pathological relevance of these mutations.

## Materials and methods

### Study subjects

Unrelated MODY probands fulfilling the classical MODY criteria (diagnosis age < 25 years old, prominent family history of diabetes spanning no less than three generations, non-obesity, and negative for diabetes-related antibodies) and their family members were referred for genetic testing and standardized clinical feature assessment at the First Affiliated Hospital of Zhengzhou University. All participants, including patients and their family members, willingly consented to take part in our precision diabetes study by providing signed informed consent. The Ethics Review Committee of First Affiliated Hospital of Zhengzhou University reviewed and approved this study. The diagnosis of diabetes, impaired fasting glucose (IFG), and impaired glucose tolerance (IGT) was based on the criteria outlined by the American Diabetes Association (2019) (13).

### Whole exome sequencing

Peripheral blood leukocytes or saliva samples were utilized for the extraction of genomic DNA by using commercially available kits (Qiagen, Hilden, Germany). We conducted WES on the probands of the suspected MODY pedigrees using the SureSelect Human All Exon V5 Enrichment kit (Agilent, USA) on the Illumina Hiseq 4000 platform (Illumina, USA). We aligned the sequencing reads to the human reference genome (hg19/GRCh37) by using the Burrows-Wheeler Aligner (BWA) tool, and removed polymerase chain reaction (PCR) duplicates by using Picard v1.57 (http://picard.sourceforge.net/). The Verita Trekker Variant Detection System using Berry Genomics and GATK software (https://software.broadinstitute.org/gatk/) was used for variant calling (14). The identified variants were categorized into the five groups based on the American College of Medical Genetics and Genomics (ACMG) guidelines for interpretation of sequence variants: “pathogenic,” “likely pathogenic,” “uncertain significance,” “likely benign,” and “benign” (15).

### Sanger sequencing

We performed PCR on each proband to verify the WES results and their affected relatives for co-segregation studies. The primers used in this study were available upon request. The PCR products were subjected to bidirectional sequencing by using the ABI3730 automated sequencer (Applied Biosystems, Foster City, CA, USA).

### Clinical and laboratory examinations

Baseline information, such as age, birth weight, history of neonatal hypoglycemia, diabetes duration, family history of diabetes, and antidiabetic therapy, was obtained by consulting the participants. The BMI was determined by dividing weight (in kilograms) by the square of height (in meters). Biochemical indexes, such as fasting plasma glucose (FPG), 2-hour plasma glucose (2hPG), triglycerides (TG), total cholesterol (TC), low-density lipoprotein (LDL), and high-density lipoprotein (HDL) cholesterol levels were assessed by a Unicel DXC800 biochemistry analyzer (Beckman Coulter). Glycated hemoglobin (HbA1c) levels were measured using the high-performance liquid chromatography (HPLC) method. Insulin and C-peptide levels were detected using an electrochemiluminescence immunoassay on a Cobas e411 analyzer (Roche Diagnostics GmbH, Mannheim, Germany). GAD and IA2 levels were measured by ELISA (SIMENS ADVIA Centaur XP, Germany). The HOMA-IR index was determined by multiplying the fasting plasma insulin level (uU/ml) with the fasting plasma glucose level (mmol/l) and dividing the result by 22.5, providing an estimation of insulin resistance. To assess pancreatic β-cell function, the HOMA-B index was computed by multiplying the fasting plasma insulin level (uU/ml) with 20, and then dividing the result by the fasting plasma glucose level minus 3.5.

### *In silico* analysis of identified variants

*In silico* prediction software, including SIFT (http://sift.jcvi.org), PolyPhen-2 (http://genetics.bwh.harvard.edu/pph2/), Mutation Assessor (http://mutationassessor.org/), MutationTaster (http://www.mutationtaster.org/), FATHMM (http://fathmm.biocompute.org.uk/), and LRT (http://www.genetics.wustl.edu/jflab/lrt_query.html), were employed to assess the functional implications of the identified missense variants. Tertiary structures of the mutated proteins were predicted using PyMOL software (https://sourceforge.net/projects/pymol/).

## Results

### Exome sequencing identifies *HNF4A* mutations from three MODY families

**Figure 1:**
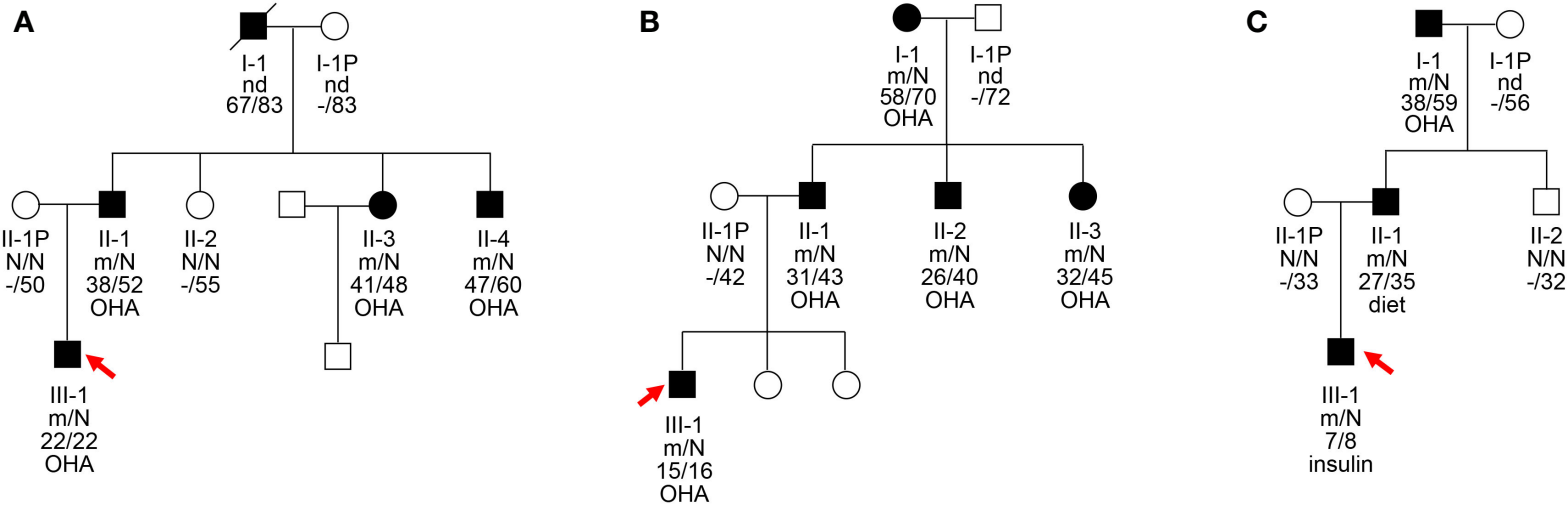
Pedigrees, genotypes, and clinical characteristics of three MODY1 families **(A–C)**. Black circles and squares, participants diagnosed with MODY1; white circles and squares, normal glucose tolerance (NGT); red arrows, probands for the three families. Numbers under the symbols are the family members’ identification numbers, followed by the genotype of mutation, then age at diagnosis of diabetes and age at examination, followed by treatment for diabetes. nd, not detected; OHA, oral hypoglycemic agents; N, normal allele; m, mutant allele.

**Figure 2:**
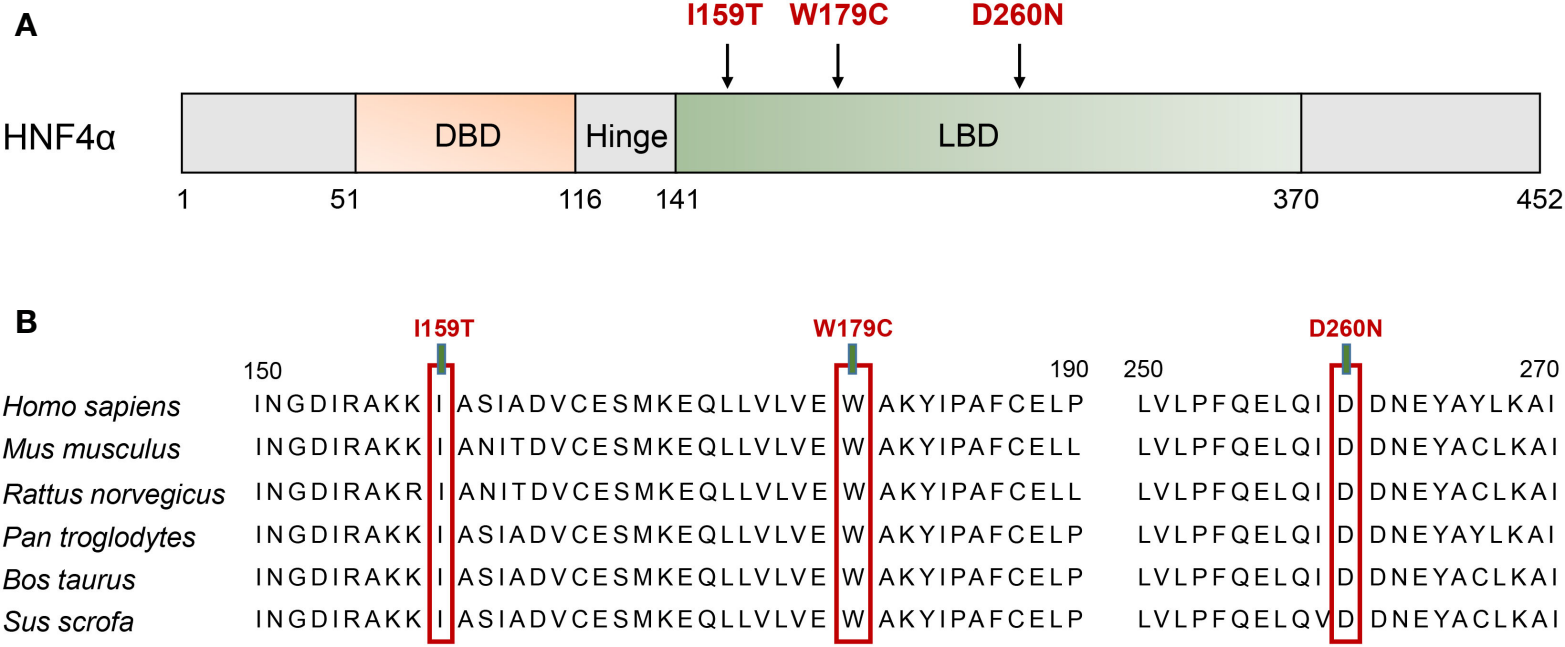
Schematic illustration of the HNF4A protein and the conservation analysis of the mutation sites. **(A)** The distribution of identified mutation among the linear HNF4A protein. The functional domains of the HNF4A protein are shown. Filled arrows indicate the mutations identified in HNF4A. DBD, DNA binding domain; LBD, ligand binding domains. **(B)** Alignment of specific regions of HNF4A from different mammals. The 150–190 aa and 250–270 aa in the first line are from human HNF4A isoform 5 (NP_787110.2, NM_175914.5).

Heterozygous missense mutations in the HNF4A gene (NM_175914.5), I159T (c.476T>C, p.Ile159Thr), W179C (c.537G>C, p.Trp179Cys), and D260N (c.778G>A, p.Asp260Asn), were identified in the probands of three unrelated MODY families, which co-segregated with hyperglycemic phenotypes in their families (**Figure 1**). The W179C mutation was identified as novel since it has not been reported in the 1000G, gnomAD, or ExAC databases. The I159T and D260N mutations were previously reported in the gnomAD database with frequencies of 1/251,466 and 1/125,742, respectively. These three mutations affected highly conserved amino acids within the LBD of HNF4α, and amino acid substitution at these sites might not be tolerated (**Figure 2**). Additionally, three mutations were predicted to be deleterious using multiple bioinformatics software packages, providing further possibilities for their pathogenicity.

### Clinical characteristics of MODY families with *HNF4A* mutations

The clinical features and biochemical parameters of the probands from the three MODY1 families are shown in **Table 1**. In family A, the I159T mutation was observed to co-segregate with diabetes, as it was present in the proband, as well as the proband’s father, paternal aunt, and paternal uncle, who were diagnosed with diabetes at the ages of 22, 38, 41, and 47, respectively (**Figure 1A** and **Table 1**). The proband was diagnosed with diabetes at 22 and complained of polydipsia and polyuria. On admission, he was prescribed low-dose insulin in combination with diet treatment owing to higher glucose levels, with an HbA1c level of 11.88%. When genetic testing was conducted and confirmatory for an *HNF4A*-MODY, the patient was tentatively switched to sulfonylureas and achieved proper glycemic control. His HbA1c level improved to 5.8% after 6 months of sulfonylureas therapy, and he reported fewer hypoglycemic events. He had no diabetic microvascular or macrovascular complications, and advanced glycation end-product products (AGE) assessing vascular complications were normal. However, the probands were born with a birth weight of 2,900 g at 39 weeks of gestation without impaired glucose tolerance. This is inconsistent with the increased birth weight and transient neonatal hypoglycemia of most *HNF4A* mutation carriers. The proband’s father, paternal aunt and uncle were treated with sulfonylureas associated with metformin shortly after the diagnosis of diabetes, and glycemic control was satisfactory. The paternal grandfather with diabetes died of lung cancer at 83, and his DNA sample was not available to check for co-segregation. The clinical characteristics of the four affected family members were indicative with a diagnosis of MODY; they were non-obese, and their diabetes was non-insulin-dependent.

**Table 1:** The genotype and phenotype characteristics of probands in three MODY families.

	Proband A	Proband B	Proband C
Nucleotide change	c.476T>C	c.537G>C	c.778G>A
Amino acid change	p.Ile159Thr	p.Trp179Cys	p.Asp260Asn
Age (years)	22	16	8
Onset age (years)	22	15	7
BMI (kg/m^2^)	22.02	24.61	16.58
Birth weight (kg)	2.9	3.3	2.45
History of neonatal hypoglycemia	No	No	No
FPG (mmol/L)	6.7	5.7	6.1
2h-PG (mmol/L)	14.7	16.3	11.4
FINS (μU/ml)	9.7	9	–
2h-INS (μU/ml)	22.3	24.5	–
Fasting C-peptide (ng/ml)	1.23	1.21	0.08
2h C-peptide (ng/ml)	4.06	4.68	–
HbA1c (%)	11.88	9.3	12.5
HOMA-IR	2.89	2.28	–
HOMA-B (%)	60.63	81.82	–
TC (mmol/L)	3.69	3.07	3.96
TG (mmol/L)	1.09	0.43	0.73
HDL-C (mmol/L)	0.79	0.94	2.03
LDL-C (mmol/L)	2.43	2.04	1.82
ALT (U/L)	17	14	15
AST (U/L)	29	14	21
GTT (U/L)	15	14	11
ALP (U/L)	26	160	185
Antidiabetic therapy	Sulfonylureas	Metformin	Insulin

BMI, body mass index; FPG, fasting plasma glucose; 2h-PG, 2-hour plasma glucose; FINS, fasting insulin; 2h-INS, 2-hour insulin; HbA1c, glycated hemoglobin; HOMA-IR, homeostasis model assessment of insulin resistance; HOMA-B, homeostasis model assessment of β-cell function. TC, total cholesterol; TG, triglyceride; HDL-C, high-density lipoprotein cholesterol; LDL-C, low-density lipoprotein cholesterol; ALT, alanine aminotransferase; AST, aspartate aminotransferase; GGT, glutamyltranspeptidase; ALP, alkaline phosphatase.

The novel mutation, W179C, was identified in a proband who was diagnosed with diabetes at the age of 15 years (BMI, 24.61 kg/m^2^) in family B (**Figure 1B** and **Table 1**). He was admitted to our hospital for the management of poor glycemic control due to self-discontinuation of metformin at the age of 16 years. The patient was diagnosed with slight diabetic retinopathy and peripheral neuropathy and was initially prescribed low-dose insulin and metformin. Given his reasonable insulin reserve with a HOMA-B score of 81.82% and higher BMI, together with the deterioration of glycemic control attributed to the withdrawal of metformin, insulin was gradually stopped after discharge, and the proband is currently receiving metformin combined with diet therapy to achieve a stable, well-controlled glycemic profile (HbA1c <6.5%). This family had three consecutive generations of diabetes, and the W179C mutation in the proband was inherited from his father with diabetes aged 31. The proband’s paternal aunt and uncle were both diagnosed with diabetes before 35 years and were confirmed to be mutation carriers. The proband’s maternal grandmother with diabetes also presented with a mutation and had diabetic retinopathy and peripheral neuropathy. All diabetic relatives in family B maintained acceptable glycemic control with metformin combined with lifestyle interventions.

The proband in family C had a low birth weight and no HH at birth (**Figure 1C** and **Table 1**). The neurological and physical development was normal. He was diagnosed with diabetes after presenting with polyuria–polydipsia at the age of seven. Considering his high blood glucose level with an HbA1c of 12.5% and decreased pancreatic islet function with fasting C-peptide <0.08 ng/ml, the proband was treated with insulin. The proband had normal liver and kidney function and no diabetes-related complications. Given that the proband had early-onset diabetes and tested negative for diabetes-related antibodies (GAD, IA2, IAA, ICA, and Zn-T8), exome sequencing was performed to identify the D260N mutation in *HNF4A*. The proband’s father, at the age of 27, was diagnosed with diabetes through an oral glucose tolerance test (OGTT) and was confirmed to be a carrier of the D260N mutation. The proband’s non-diabetic mother did not carry this mutation. The paternal and maternal grandfathers of the proband were diagnosed with diabetes at 38 and 53, respectively. They initially managed the condition with dietary changes but were subsequently started on metformin. Cascade genetic screening revealed the presence of the same mutation in the proband’s paternal grandfathers but not in his maternal grandfathers, confirming a paternally derived mutation and autosomal dominant inheritance in this family.

### *In silico* simulation analysis

**Figure 3:**
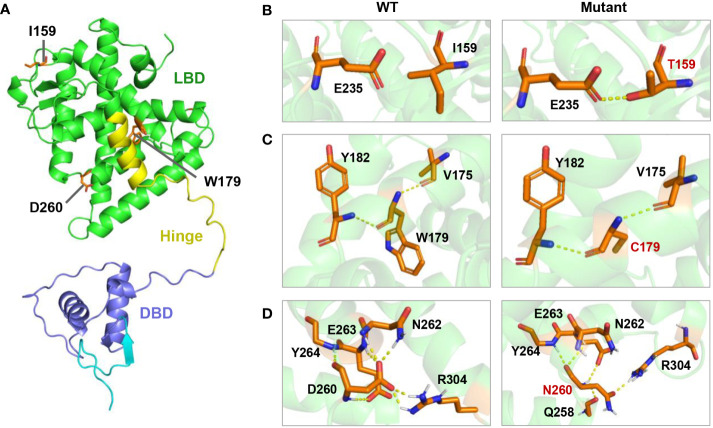
Molecular model of I159T,W179C, and D260N mutation of HNF4A in PyMOL. **(A)** Location of three mutations in the three-dimensional model of human HNF4A protein. **(B)** The I159T mutation led to a new hydrogen bond between T159 and E236. **(C)** The W179C mutation introduced a smaller side chain of amino acid. **(D)** D260N substitution resulted in the loss of the hydrogen bonds formed at D260 with N262 and R304.

By employing *in silico* simulations, we generated the three-dimensional structures of both the wild-type *HNF4A* and its mutants (I159T, W179C, and D260N) (**Figure 3**). As shown in **Figure 3A**, the location of the three *HNF4A* mutations was mapped on the global landscape of the HNF4A protein (**Figure 3A**). The I159T mutation altered a residue positioned on the LBD, substituting a non-polar hydrophobic I159 residue with a polar hydrophilic T159 residue. Molecular modeling of the *HNF4A*-I159T mutation in PyMOL predicted that the mutated T159 formed a pair of new hydrogen bonds with the E235 residue (**Figure 3B**). The W179C mutation, a non-polar hydrophobic W179 substituted by a polar hydrophilic residue, C179, may affect the local structure of the protein owing to the altered side chain, although the distribution of the surrounding charge was unaffected (**Figure 3C**). For the D260N mutation in the LBD, negatively charged D260 was substituted with uncharged N260, probably weakening the interaction with the surrounding residues. As predicted by PyMOL, D260 formed hydrogen bonds with the surrounding residues N262, E263, Y264, and R304 in the wild-type HNF4A protein, while the D260N substitution resulted in the loss of hydrogen bonds with N262 and R304, suggesting a weakened interaction (**Figure 3D**).

## Discussion

*HNF4A*/MODY1 mutations are frequently identified in Caucasian populations, accounting for an estimated 5% of MODY cases, whereas *HNF4A* mutations are rarely reported in Han Chinese families (16–18). Fewer than 20 *HNF4A* mutations have been described in the Chinese MODY pedigrees (16–18). This study identified the I159T, W179C, and D260N mutations in *HNF4A* that co-segregated with the diabetic phenotype in three suspected MODY pedigree cases, broadening the spectrum of MODY1-associated *HNF4A* mutations (**Figure 1**). Molecular model predictions of HNF4A mutants in PyMOL showed that the I159T and D260N mutations altered polar interactions with surrounding residues, and W179C resulted in a smaller side chain, suggesting an altered structure of the mutant proteins (**Figure 3**). Therefore, pedigree segregation, population data, and *in silico* simulation analysis provided pathogenic evidence for the identified mutations responsible for the diabetes phenotype in these three families.

Heterozygous mutations in *HNF4A* can give rise to biphasic manifestations, characterized by either persistent or transient HH in infants and MODY1 in young adults. Fifty-six percent of *HNF4A* mutation carriers have been reported to have macrosomia that always occurs in neonatal HH (19). The two probands in this study had normal birth weights and no experience of infant hypoglycemia, whereas the proband in family C had a lower birth weight. The risk of macrosomia is reported to be higher in maternally inherited mutations than in paternally inherited mutations due to the additional effect of the intrauterine environment on hyperglycemia (19). The paternal inheritance pattern in our three pedigree cases may explain the absence of macrosomia in their offspring. In a recent cohort study, it was observed that individuals with an *HNF4A* mutation and higher birth weight exhibited a lower decline in adult beta cell function. Birth weight was identified as a potential modifier with prognostic and therapeutic relevance in this context (10). Thus, the proband in family C diagnosed with diabetes at 7 years old and had weakened insulin secretory capacity may be associated with low birth weight. Alternatively, in addition to the paternally inherited *HNF4A* mutation, the T2DM susceptibility gene from the proband’s maternal grandfather may contribute to the severe clinical phenotype of this proband. In addition, the clinical phenotype of MODY1 varied in patients with different *HNF4A* mutations and even within the family members carrying the same mutation, and the younger generation in multigenerational families frequently exhibited an earlier age of onset and more severe phenotypes (5, 11, 12). Age-related penetrance of diabetes has been reported; for example, by the age of 40 years, 98% of probands and 76% of family members developed diabetes, and by the age of 50 years, 99% of probands and 90% of family members developed diabetes (20). In this study, probands were all diagnosed with diabetes before 25 years old, and the proportion of family member carriers developing diabetes was 67% (6/9) by 40 years and 89% (8/9) by 50 years, which is consistent with the high penetrance reported in clinically confirmed MODY cohorts (20).

The molecular diagnosis of MODY has substantial implications for the management of diabetes, and individuals with *HNF4A*-MODY are optimally treated with low-dose sulfonylureas (21). However, not all *HNF4A*-MODY individuals could achieve successful transfer to sulfonylurea treatment, and shorter diabetes duration and lower level of BMI and HbA1c at the time of genetic diagnosis have been identified as predictors of successful treatment with sulfonylureas or diet alone in those who have *HNF4A*-MODY (22). The proband in family A, with a normal BMI, whose genetic etiology was confirmed following the diagnosis of diabetes, was successfully converted to sulfonylureas and achieved good glycemic control. The proband of family B responded well to metformin combined with diet therapy due to a higher BMI; therefore, we did not transfer sulfonylureas from metformin. A prospective national study found that only 36% of individuals with *HNF1A/HNF4A*-MODY who changed treatment to sulfonylureas/diet alone achieved good glycemic control (22). The proband in family C with paternal-derived mutations had markedly defective pancreatic β-cell function due to the superposition effect of the maternal grandfathers’ T2DM susceptibility genes, and we did not attempt to switch to sulfonylurea therapy for this proband. A recent study demonstrated the successful use of GLP-1 receptor agonist therapy in a father–son MODY1 cohort in which the son was switched to semaglutide after glimepiride failure. At the same time, the father gradually discontinued insulin and switched to liraglutide (23). Treatment with glimepiride or liraglutide in *HNF1A*-MODY patients can achieve equivalent FPG and postprandial glucose excursions, and liraglutide has a lower risk of mild hypoglycemia (24). Activation of GLP-1 receptors on β cells induces the stimulation of adenylate cyclase, leading to an increase in cAMP levels. This mechanism is believed to bypass the reduced ATP concentrations observed in *HNF1A*-MODY and *HNF4A*-MODY, resulting in the stimulation of insulin secretion and the reduction of postprandial glucose levels (23, 24). In addition, liraglutide was reported to be effective in controlling plasma glucose levels in patients with ABCC8 mutations who were sensitive to high doses of sulfonylureas (25). Therefore, GLP-1 receptor agonist (GLP-1 RA) could be an effective therapy to consider in *HNF4A*-MODY patients with sulfonylureas failure or requiring discontinuing insulin.

Three missense mutations identified in this study affected the highly conserved residues in the LBD of HNF4α and altered the interaction with surrounding residues and side chain (**Figures 2**, **3**). The D260N mutation resulted in more significant alterations in amino acid interactions compared to I159T and W197C, partly explaining the low insulin secretion observed in proband C. Moreover, the D260N mutation positioned in the LBD is spatially close to the DBD, potentially compromising DNA binding capacity in addition to its effect on decreased transcription activity. A previous study has shown that *HNF1A*-T260M mutation led to severe damage to DNA binding and transcription activity due to disrupting the hydrogen bond formed with R263 (26). Functional studies based on human islets have also confirmed the defective transcriptional regulatory networks of islets β cell caused by this mutation (26). Consistently, Chandra et al. performed structural analysis on the crystal structure of the obligate homodimer HNF4α in complex with its DNA element and coactivator-derived peptides. The results revealed the physical and functional integration of the LBD and DBD modules, enabling the formation of a strong and specific interaction with DNA (8). Moreover, certain mutations associated with MODY1 located within the LBD compromise DNA binding by interacting with the inter-junctional surfaces of the complex (8). Therefore, the identified mutations in the LBD may reduce the receptor’s DNA affinity and transcriptional activity, requiring further wet experiments for confirmation.

Previous studies found that complete deletion of *HNF4A* in mice leads to early embryonic death, and specific deletion of *HNF4A* in mouse pancreatic β cells does not cause diabetes phenotype, despite impaired glucose-stimulated insulin secretion (GSIS) (27, 28). These findings suggest that rodent models do not fully replicate the phenotypic characteristics of human MODY1. Recently, human-induced pluripotent stem cell (hiPSC)-based disease modeling strategies have provided novel and successful opportunities for investigating the pathogenesis of monogenic diabetes (29–31). A recent hiPSC-based study of the *HNF4A*-Ile271fs mutation showed that in individuals with MODY1, there are early disruptions in liver and pancreas development, which contribute to altered hepatic proteins and defects in β cells among patients, and defective β-cell function led to the loss of transcriptional activation of target genes by mutant *HNF4A* (32). Clinically, in addition to progressive β-cell insulin secretion defects, some MODY1 patients also exhibit mild liver abnormalities, such as low serum levels of triglycerides, HDL-C, and apolipoproteins (3, 33). Three probands carrying *HNF4A* missense mutation in this study showed variable pancreatic β-cell dysfunction, and the proband in family A had decreased HDL-C level (**Table 1**) and hepatic cyst as seen in image diagnostics (data not shown). However, whether these mutations identified in our study lead to the MODY1 phenotype in humans via a similar mechanism remains less clear-cut. Further hiPSC-based studies on *HNF4A* missense mutations can offer novel perspectives on the pathogenic mechanism of MODY1, given that most of the currently identified MODY1-related *HNF4A* mutations are always of the missense type (5).

In conclusion, we described three heterozygous missense mutations in *HNF4A* in Chinese MODY families, expanding the mutation spectrum of *HNF4A*. Pedigree segregation, clinical features, and *in silico* simulation analysis provided pathogenic evidence of the identified mutations responsible for the diabetes phenotype in these three families. Molecular diagnosis of these patients is vital because of the implications for precision therapy, pregnancy, and screening of at-risk family members.

## Data availability statement

The original contributions presented in the study are publicly available. This data can be found here: ClinVar database (https://www.ncbi.nlm.nih.gov/clinvar/), accession numbers VCV002574632, VCV002574633, VCV002574634.

## Ethics statement

The studies involving humans were approved by The Ethics Review Committee of First Affiliated Hospital of Zhengzhou University. The studies were conducted in accordance with the local legislation and institutional requirements. Written informed consent for participation in this study was provided by the participants’ legal guardians/next of kin. Written informed consent was obtained from the individual(s), and minor(s)’ legal guardian/next of kin, for the publication of any potentially identifiable images or data included in this article.

## Author contributions

JZ, YJ, and LYa conceived and designed the study. YJ and JL performed the experiments. JZ, LYi, and YY analyzed the data. JZ and YJ wrote the manuscript. JZ, LYa, and HZ revised the manuscript. All the authors contributed to the manuscript and approved the submitted version.
